# Traumatic Aortic Dissection as a Unique Clinical Entity: A Single-Center Retrospective Study

**DOI:** 10.3390/jcm12247535

**Published:** 2023-12-06

**Authors:** Qingwei Gang, Yu Lun, Liwei Pang, Xinyang Li, Bingchen Hou, Shijie Xin, Jian Zhang

**Affiliations:** Department of Vascular and Thyroid Surgery, The First Affiliated Hospital of China Medical University, Shenyang 110001, China; qwgang@cmu.edu.cn (Q.G.); ylun@cmu.edu.cn (Y.L.); 2021120988@cmu.edu.cn (L.P.); 2023110304@cmu.edu.cn (X.L.); bchou@cmu.edu.cn (B.H.); sjxin@cmu.edu.cn (S.X.)

**Keywords:** aortic dissection, traumatic, stanford type B, blunt aortic injury, thoracic endovascular aortic repair (TEVAR)

## Abstract

Background: This study aimed to compare the clinical characteristics, treatment approaches, and outcomes of the Stanford Type B traumatic aortic dissection (TAD) with non-traumatic aortic dissection (NTAD), and assess better management for TAD. Methods: We retrospectively analyzed patients who underwent thoracic endovascular aortic repair for Stanford type B aortic dissection at The First Hospital of China Medical University between 2014 and 2022. The patients were divided into TAD and NTAD groups based on whether they had a history of acute trauma. This study ultimately included 65 patients with TAD and 288 with NTAD. We assessed and compared the baseline characteristics, laboratory indicators, imaging features, surgical procedures, and follow-up results between the groups. Results: The TAD group was younger compared to the NTAD group (50.00 [IQR40.00–59.00] vs. 55.00 [IQR 47.00–61.00] years, *p* = 0.020). A lower percentage of the TAD group had a history of hypertension (20% vs. 71.18%, *p* < 0.001). The length of aortic dissection was shorter in the TAD group compared to the NTAD group (30.00 [IQR 22.00–40.00] vs. 344.00 [IQR 237.25–400.00] mm, *p* < 0.001). All patients with TAD underwent TEVAR following the same strategy as NTAD. The mean preoperative duration was 7.00 (IQR 2.00–14.00) days in the TAD group and 11.00 (IQR 8.00–15.00) days in the NTAD group (*p <* 0.001). TAD showed fewer complications after TEVAR in mid-to-long-term follow-up. Conclusions: TAD is distinct from NTAD. TAD typically presents with more localized lesions than NTAD, and the patients experience a shorter preoperative duration and a better mid-to-long-term outcome.

## 1. Introduction

Aortic dissection (AD) is a critical vascular emergency with life-threatening implications, whose mortality rate can reach as high as 90% without prompt intervention [1,2,3]. Traumatic aortic dissection (TAD) is a specific type of AD predominantly caused by trauma and stress to the chest or back during traffic accidents or falls from heights, necessitating urgent and accurate diagnosis and treatment [4]. Traumatic aortic injuries have been categorized into four grades: intimal tear, intramural hematoma, aortic pseudoaneurysm, and free rupture [5,6]. However, TAD, as a unique form of traumatic aortic injury, has not been included or described within this classification. Despite sharing similar morphological features with non-traumatic aortic dissection (NTAD), TAD potentially represents a distinct clinical entity in terms of its mechanism, morphology, and clinical outcome, among other factors [7]. With the advancements in endovascular techniques, thoracic endovascular aortic repair (TEVAR) has emerged as an effective treatment option for AD. An increasing number of patients with aortic trauma, including TAD, are subjected to TEVAR despite the ongoing debate about the optimal intervention time. However, there is a scarcity of reports on the TEVAR treatment in patients with TAD. Therefore, this study aimed to analyze and compare the clinical data of TAD with NTAD, evaluate the clinical features and mid-to-long-term results in the TAD group after TEVAR, and provide better management for TAD.

## 2. Materials and Methods

### 2.1. Study Cohort

**Figure 1:**
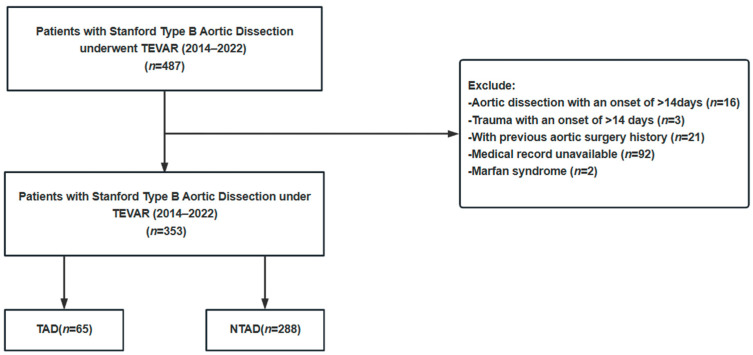
Study flowchart. This study flowchart was used to describe the inclusion process of patients diagnosed with aortic dissection and treated with thoracic endovascular aortic repair (TEVAR) between 2014 and 2022. Based on the inclusion and exclusion criteria, the patients were divided into the traumatic aortic dissection (TAD) group and the non-traumatic aortic dissection (NTAD) group.

We conducted a retrospective analysis of 487 patients diagnosed with Stanford type B AD and treated with TEVAR at The First Hospital of China Medical University from January 2014 to December 2022. All the patients underwent computed tomography angiography (CTA) for diagnosis confirmation, with the general exclusion criteria being persistent symptoms > 14 days, unavailable medical records, history of aortic diseases, connective tissue disorders, systemic inflammatory diseases, and patients < 18 years old. Among the 487 patients, exclusions were made for patients with >2 weeks of symptoms (*n* = 19), a history of aortic surgery (*n* = 21), incomplete data (Imaging data of the aorta, *n* = 92), or Marfan syndrome (*n* = 2). Ultimately, 353 patients were enrolled and divided into the TAD and NTAD groups based on acute trauma history (65 in the TAD group and 288 in the NTAD group), as depicted in Figure 1. The study was conducted in accordance with the Helsinki Declaration and approved by the Ethics Committee of The First Hospital of China Medical University, at the same time, to uphold patient privacy rights, and all the patients were presented anonymously.

### 2.2. Treatment and Follow-Up

All the patients underwent TEVAR, during which an endovascular stent graft was conventionally positioned from the arch distal to the left subclavian artery onto the descending aorta to seal the primary entry tear (oversize from 0 to 10%). Patients underwent follow-up CTA at 3, 6, and 12 months post-discharge, and subsequently on an annual basis to monitor symptoms and stent-related complications [8,9].

### 2.3. Data Measurements

Patient data were retrieved from the Department of Vascular Surgery databases at The First Hospital of China Medical University. Patient characteristics, including sex, age, body mass index (BMI), comorbidities, and injury details, were collected. Two vascular surgeons independently evaluated the image analysis for each patient. In case of disagreement, a senior vascular surgeon reviewed and confirmed the final diagnosis. The zone of AD was defined according to the criteria outlined by the Society for Vascular Surgery (SVS) and Society of Thoracic Surgeons (STS) in 2020 [10], which divided the area into 12 zones (zone 0 to zone 11), spanning from the ascending aorta (AA) to the external iliac artery. Computed tomography (CT) was utilized to determine imaging findings, such as lumen type, length, and dissection location. A 3mensio Vascular (Pie Medical Imaging BV, Bilthoven, the Netherlands) was used to reconstruct the CT images and measure along the central lumen line of the aorta. The length of dissection was defined as the distance from the distal to the proximal end of the dissection along the central axis of the aorta.

### 2.4. Statistical Analysis

Statistical methods were predetermined and detailed in an approved protocol. Data quality measures were implemented, including removing outliers and missing values, normality and variance checks, and linear relationship assessments.

In this study, we kept a detailed record of variables with missing data. We first identified which variables had missing data and calculated the number and corresponding percentage of missing data for each variable. Regarding the handling of missing data, if the missing proportion was ≤5%, we used data imputation methods (median imputation) to fill in the missing values. If the missing proportion exceeded 5%, we excluded that specific set of data. Through these approaches, we aimed to minimize the potential biases or distortions that missing data could cause.

Quantitative variables were presented as mean ± SD or median (interquartile range), while frequencies and percentages described qualitative variables. Continuous variables were compared using the *t*-tests or ANOVA, and categorical variables were compared using chi-square tests, and Fisher’s exact test. All-cause mortality was analyzed with the Kaplan–Meier method, with hazard ratios (HRs) compared using the Cox model. Visual inspection was employed by comparing line graphs between distinct groups and assessing the smoothness and variability of the curves to assess the proportional hazard assumptions for the Cox regression analyses, which allowed for determining the validity of the proportional hazard assumption. Statistical significance was set at *p* < 0.050. The software used for analysis included R (version 3.6.1, R Core Team, Vienna, Austria) and SPSS (version 26, IBM Corp, Armonk, NY, USA). The graphs were generated using GraphPad Prism 8 (GraphPad Software, La Jolla, CA, USA).

## 3. Results

### 3.1. Patient Characteristics

Among the initial cohort of 353 patients, male predominance was observed. Supplementary Table S1 provides the trauma causes and primary symptoms in all 65 TAD cases. Compared to the NTAD group, the patients in the TAD group are younger (50.00 [IQR40.00–59.00] vs. 55.00 [IQR 47.00–61.00] years, *p* = 0.020), a reduced prevalence of hypertension (20. 00 vs. 71.18%, *p* < 0.001), and lower rates of smoking (20.00% vs. 36.81%, *p* = 0.010) compared to the NTAD group. Further details are presented in Table 1.

### 3.2. Comparison of Laboratory Results

Upon admission, the TAD group presented significantly lower levels of red blood cells (RBC), hemoglobin (HGB), total cholesterol (Tc), low-density lipoprotein cholesterol (LDL-C), high-density lipoprotein cholesterol (HDL-C), homocysteine (Hcy), cystatin C, serum creatinine, and fibrinogen (FIB) (*p* < 0.050). However, the TAD group exhibited considerably higher median D-dimer levels (10.51 [IQR 4.60–18.20] vs. 4.44 [IQR 2.29–9.16] mg/L, *p* < 0.001) and a higher white blood cell count (13.04 [IQR 10.43–17.42] vs. 11.28 [IQR 8.94–13.90] × 10^9^/L, *p* < 0.001) when compared to the NTAD group. No significant differences were detected in other laboratory results (*p* > 0.050) (Table 2).

### 3.3. Radiological Findings

**Figure 2:**
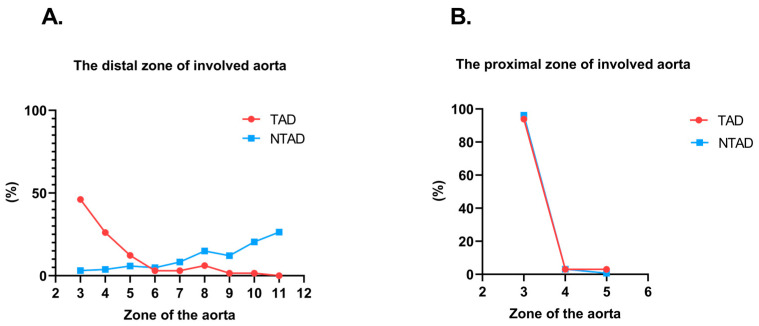
(**A**). Comparison of the distribution of distal involvement of aortic dissection between different zones of the aorta in two groups (*p* < 0.001). (**B**). Comparison of the distribution of proximal involvement of aortic dissection between different zones of the aorta in two groups (*p* = 0.228).

We analyzed the 3D CT morphological characteristics of AD in both TAD and NTAD groups (Table 3). From the perspective of aortic arch branching type, the proportion of type III arch in the TAD group was significantly lower than that in the NTAD group (6.15% vs. 18.4%, *p* = 0.008). The TAD group exhibited a shorter length along the long axis (30.00 [IQR 22.00–40.00] vs. 344.00 [IQR 237.25–400.00] mm, *p* < 0.001), indicating a lesser extent of lesion involvement. In contrast, the NTAD group displayed significantly more extensive involvement of the distal aortic dissection zone (*p* < 0.001); no difference was observed in the proximal zone (*p* = 0.228), as demonstrated in Figure 2. Moreover, the TAD group presented a smaller diameter in the ascending aorta and a lower percentage of ascending aorta diameter > 40 mm (36.86 ± 4.75 vs. 40.19 ± 4.81 mm, *p* < 0.001; 20.00% vs. 48.96%, *p* < 0.001) compared to the NTAD group. In comparison to the NTAD group, the TAD group had a larger true lumen diameter (22.40 [IQR 16.60–26.70] vs. 19.10 [IQR 15.47–23.92] mm, *p* = 0.006) and smaller false lumen diameter (16.40 [IQR 11.60–21.50] vs. 17.75 [IQR 14.40–23.53] mm, *p* = 0.037), moreover, a smaller diameter in the combined total diameter of the two lumens (35.80 [IQR 32.10–39.80] vs. 37.70 [IQR 34.00–42.02] mm, *p* = 0.013). The TAD group had a significantly higher proportion of dissections originating on the lesser curve (44.62% vs. 23.96%, *p* = 0.001) and a lower proportion of multiple entry tears (3.08% vs. 29.51%, *p* < 0.001) and complicated aortic dissection (0% vs. 7.29%, *p* = 0.001) than the NTAD group. No significant differences were observed in other characteristics between the two groups. Please refer to Table 3 for specific results.

### 3.4. Surgical Strategy

The treatment duration for TAD primarily depended on the patient’s hemodynamic stability related to the aortic lesion. An unstable hemodynamic state, characterized by increasing pleural effusion, ongoing chest pain, and signs of rupture, indicated the need for an emergent intervention. Patients with hemodynamically stable TAD typically receive interventions in the sub-acute phase, similar to most patients with NTAD. In the TAD group, four patients (6.15%) underwent urgent treatments within 24 h after TAD onset, and 15 (23.08%) received acute treatments within 48 h after TAD onset. Consequently, patients in the TAD group had a shorter preoperative time than those in the NTAD group (7.00 [IQR 2.00–14.00] vs. 11.00 [IQR 8.00–15.00] days; *p* < 0.001).

The TAD group had a smaller average stent diameter (32.00 [IQR 28.00–34.00] vs. 32.00 [IQR 30.00–34.00] mm; *p* = 0.020) and a shorter stent length (160.00 [IQR 150.00–180.00] vs. 180.00 [IQR 160.00–200.00] mm; *p* < 0.001) compared to the NTAD group. However, other perioperative details did not exhibit significant differences between the two groups (*p* > 0.050) (Table 4).

### 3.5. Perioperative Complications

Although 14 patients (21.54%) in the TAD group and 66 (22.91%) in the NTAD group developed perioperative complications, the difference was not significant (*p* = 0.849) (Table 5). In the TAD group, 14 patients experienced various adversities, including two (3.08%) with systemic inflammatory response syndrome who succumbed despite intensive treatment, one (1.54%) with kidney ischemia who responded well to vasodilators and renal function-enhancing drugs, four (6.15%) with puncture site hematoma which resolved gradually with local pressure bandages, five (7.69%) with deep vein thrombosis who received anticoagulant therapy, and two (3.08%) with pneumonia who showed significant symptom improvement after receiving active anti-inflammatory and expectorant treatments. The NTAD group had a similar incidence of perioperative complications, including SIRS (1, 0.35%), kidney ischemia (2, 0.69%), endoleak (34, 11.80%), and puncture site hematoma (29, 10.07%). Unfortunately, the patient with SIRS in the NTAD group died despite active treatment. Type I endoleak occurred in 34 patients during surgery, and stents were placed at the proximal end of the dissection in eight cases. In the remaining 26 cases, observation without intervention was deemed sufficient due to mild endoleak. Secondary renal ischemia and puncture site hematoma improved with active treatment.

### 3.6. Mid-to-Long Term Outcomes

In this study,353 patients completed the follow-up study, with 48 patients lost to follow-up, resulting in an approximate loss rate of 13.60%. The duration of follow-up was comparable between the TAD and NTAD groups (36.00 [IQR 18.00–56.0] vs. 40.00 [IQR 24.00–62.50] months, *p* = 0.683). Three cases of retrograde dissection (1.04%) of the aortic dissection after TEVAR, with one patient undergoing the chimney technique and endovascular stent implantation. Long-term follow-up revealed significantly superior outcomes in the TAD group compared to the NTAD group (0.00% vs. 13.19%, *p* = 0.004) (Table 5). Four patients (1.39%) developed pseudoaneurysms requiring reintervention with the TEVAR procedures. Three patients’ pseudoaneurysms resulted from proximal endoleak, while the other suffered from distal stent-graft-induced new entry (SINE), leading to a rupture of a distal pseudoaneurysm. One patient declined further surgery for distal SINE encompassing the stent. Upper limb ischemia due to left subclavian artery (LSA) coverage was observed in two patients (0.69%) who underwent fenestration reconstruction. Three patients (1.04%) with severe lower limb ischemia experienced significant symptom improvement after successful embolus removal. Two patients (0.69%) developed renal impairment or failure, with one requiring emergency hemodialysis. Intestinal ischemia occurred in one patient (0.35%), leading to intestinal resection. Furthermore, 19 patients (6.61%) displayed distal segmental aortic enlargement during follow-up; however, they remained asymptomatic and were closely monitored.

**Figure 3:**
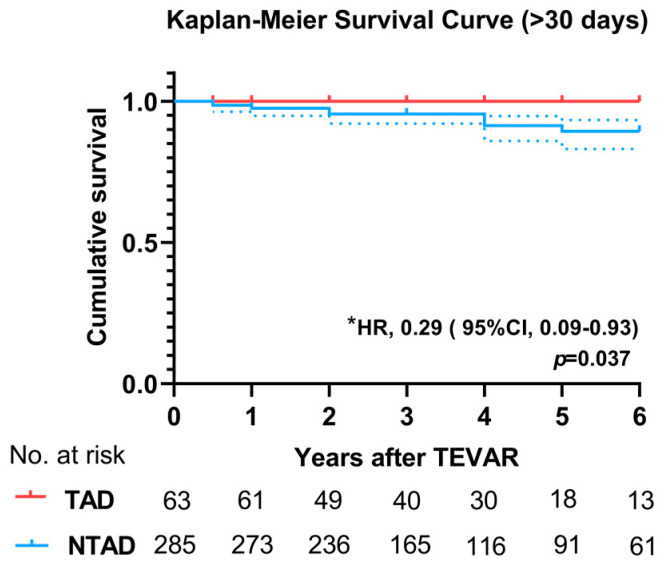
Kaplan–Meier survival curves for mid-to-long-term postoperative outcomes between the TAD and NTAD groups. Average follow-up time: 3.04 years in TAD, 3.33 years in NTAD. * The mid-to-long-term mortality rate in the TAD group is significantly lower than in the NTAD group.

The Kaplan–Meier method and log-rank survival test were employed to analyze survival data. The *p*-values were 0.037, as shown in Figure 3. A significant difference was observed in the risk of complications (OR, 0.49; 95% CI 0.26–0.92; *p* = 0.027), although not in the risk of mortality (HR, 0.38; 95% CI 0.08–1.68; *p* = 0.203). (Table 6).

## 4. Discussion

To the best of our knowledge, this is the first clinical study for TAD up to date. In our study, we found that TAD is distinct from NTAD. Compared to patients with NTAD, TAD are younger and have a lower proportion of hypertension, a shorter length of dissection and shorter preoperative duration, etc. During the long-term follow-up, the TAD group exhibited a lower incidence of complications and long-term mortality rates following TEVAR than the NTAD group.

Aortic blunt injury frequently results from external forces applied to the chest during traffic accidents or falls from heights, leading to aortic trauma of varied severity [11]. Aortic trauma can lead to AD through intimal tears; however, the exact mechanism remains unknown. In contrast, NTAD, or spontaneous AD, is caused by high blood pressure acting on the aortic wall, leading to intimal tears. Risk factors for NTAD include genetics, smoking, hypertension, and atherosclerosis [12,13,14]. Hypertension is a significant risk factor for predisposing to AD by subjecting the aortic wall to increased shear stress [15]. The International Registry of Acute Aortic Dissection (IRAD), which is a consortium of 58 research centers in 13 countries, was established in 1996 and evaluates the management and outcomes of acute aortic dissection and intramural hematoma, assessing etiological factors, clinical features, treatment, and post-discharge outcomes globally, reported that 74.4% of them had a history of hypertension at least [13,16]. Hypertension contributes to NTAD by triggering spontaneous aortic intimal tears, indicating an underlying pathological condition or their own vascular characteristics, such as type III aortic arch, a radiological feature known to increase the risk of type B aortic dissection (TBAD) [17]. Meanwhile, age is also a significant factor in the occurrence of aortic dissection. Data from IRAD suggests that the average age of patients with acute type B aortic dissection is 63.6 ± 14.1 years, another data from Germany over 9 years reported an average age of 66 [IQR 56–74] years for patients with TBAD [16,18]. Our results showed an age of 55 [IQR 47–61] years for patients with NTAD and 50 [IQR 40–59] years for the TAD group, Chinese patients are younger, which is consistent with previous research findings [19]. Meanwhile, as a risk factor for aortic dissection, aortic root diameter increases with age [20]. Animal experiments have demonstrated that aortic medial degeneration leads to age-dependent aortic dilation and, under hypertensive stress, results in aortic dissection [21]. In our study, patients with TBAD who underwent TEVAR surgery in the NTAD group had a higher prevalence of smoking, hypertension type III aortic arch, and an enlarged ascending aorta compared to the TAD group.

Patients with NTAD had a longer dissection length and a larger maximum diameter of the ascending aorta compared to the TAD group, with a significant proportion (up to 48.96%) having a maximum ascending aorta diameter of >40 mm. Similarly, more aortic zones were involved in the NTAD group than in the TAD group. Our results corroborate findings from the IRAD, which reported a high prevalence (40.2%) of ascending aorta diameters >40 mm in patients with NTAD. However, the all-cause mortalities did not differ based on the diameter size of the ascending aorta in their study [22]. The differences in ascending aorta diameter are likely attributed to the long-term effects of hypertension or arteriosclerosis. In evaluating the dissection features in the TAD group, we analyzed the prevalence of dissection on the lesser curvature side, multiple channels, the maximum diameter of the true/false lumen, and complicated aortic dissection. Contrastingly, we found that TAD more frequently extended from the lesser curvature of the aortic arch, while NTAD was more prone to having multiple tears. The anatomic features of the aorta could explain these differences. The relatively mobile aortic arch and the fixed descending aorta create a vulnerable aortic isthmus between them, which is more susceptible to various mechanical forces. Therefore, the PETs are ideally positioned in the aortic isthmus, particularly in the lesser curvature. This finding aligns with previous research [23]. The inherent vulnerability of the aorta to any injury in NTAD induces blood to strike the arterial wall, causing tears in the aortic isthmus between the aortic arch and the descending aorta. Blood from the aortic lumen enters the aortic media through these intimal tears, resulting in perfused channel formation in the aortic media. These channels expand along the long axis of the aorta, creating a new lumen within the aortic wall [24].

Interestingly, no significant difference was observed in the distance between the LSA and the PET between the two groups, indicating that the most common site for the primary tear is at the junction of the arch and descending aorta, where the curvature alters its direction, affecting the blood flow shear. This anatomical and structural vulnerability renders the aorta susceptible to injuries from external and internal forces. However, a significantly higher proportion of dissections originate on the lesser curve, possibly suggesting differences in internal and external forces and the anatomical characteristics of the aortic arch.

With recent advancements in endovascular technology, TEVAR has emerged as a favorable treatment option for blunt thoracic aortic injury (BTAI) compared to open aortic repair [25]. Regarding perioperative outcomes, our study found no significant differences between the two groups regarding surgical duration, the mean number of stents implanted per patient, and TEVAR-assistive techniques. However, the optimal timing for intervention remains uncertain. The 2011 clinical practice guidelines from the SVS suggest urgent repair (≤24 h) for stable aortic trauma unless accompanied by other non-aortic serious injuries [26]. However, a retrospective observational study involving 548 patients compared early (≤24 h) versus delayed (>24 h) TEVAR for BTAI. The study indicated that delayed TEVAR was associated with a lower mortality risk, even after adjusting for the aortic injury grade [27]. As for TAD, there is currently no recommended optimal timing for intervention. According to a recent meta-analysis, TEVAR has shown a favorable long-term prognosis for blunt aortic trauma, with rare instances requiring reinterventions [28]. The American College of Cardiology (ACC) and the American Heart Association (AHA) 2022 guidelines for the Diagnosis and Management of Aortic Disease indicate that the timing of surgical intervention remains unclear [6]. In our center, the intervention time is based on hemodynamical stability and an unstable state with signs of aortic rupture necessitating an urgent intervention. For others, we follow a conventional treatment strategy for NTAD, with a mean preoperative duration of 11 days. Although the patients with TAD had a four-day shorter preoperative waiting time, we did not observe significant differences in complications, all-cause mortality, and survival time. However, the proportion of complications and deaths in the TAD group was lower, with mortalities primarily resulting from non-aortic-related causes. Table 6 presents the incidence risks of complications and mortalities in the TAD and NTAD populations. Although there was no significant difference in early mortality between the two groups, the TAD group showed significantly better in terms of mid-to-long-term outcomes. We primarily attribute this outcome to the trauma associated with the TAD group, while the differences in long-term complications and mortality rates may be due to factors such as the extensive involvement of the dissection in the NTAD group and a higher proportion of complex dissections.

D-dimer concentration strongly correlates with AD and has been suggested as a parameter to predict outcomes and rule out or confirm AD in patients [29,30,31]. In our study, patients in the TAD group had significantly higher median D-dimer levels. We hypothesize that the elevated D-dimer levels in the TAD group may be attributed to trauma-induced imbalances between plasminogen activators and inhibitors. Therefore, we conclude that D-dimers could not fully predict the clinical outcome in this study. Meanwhile, the TAD group experienced significant reductions in RBC and HGB levels, possibly due to blood loss directly related to the trauma. The reduction in Hb level and also the higher level of D-dimer might be related not only to TAD. Being TAD is usually related to other trauma, the blood loss and D-dimer might reflect also other sites of injury. Plasma levels of Hcy are independent risk factors for severe cardiovascular involvement and AD [32]. Our study confirmed that the plasma homocysteine level was significantly lower in the TAD group compared to the NTAD group. As NTAD is closely associated with hypertension and is also suspected of having underlying structural defects, this partly explains why the maximum diameter of the false lumen and the total diameter of the false lumen plus the true lumen is larger in NTAD than in TAD, as observed in our study.

Our study has several limitations: a small number of patients in the TAD group, which limits the generalizability of our prognostic study. Additionally, this is a single-center, retrospective study, there may be a selection bias, leading to a non-random and non-comprehensive sample, which also raises concerns about low external validity; retrospective studies rely on patient recall or medical records. Additionally, there may also be a recall bias.

## 5. Conclusions

Our study compared and analyzed the clinical manifestations, imaging features, operative characteristics, and prognosis of patients with TAD and NTAD. We found that TAD is distinct from NTAD. TAD typically presents with more localized lesions, and the patients experience a shorter preoperative duration and a better mid-to-long-term outcome. Further research is necessary and can be achieved by increasing sample size and fostering collaboration among multiple centers. If possible, investigate and compare the pathological traits of TAD and NTAD through basic research.

## Figures and Tables

**Table 1:** Characteristics of patients in the TAD and NTAD groups.

Parameters	TAD (*n* = 65)	NTAD (*n* = 288)	*p* Value
Sex			0.906
Female	12 (18.46)	55 (19.10)	
Male	53 (81.54)	233 (80.90)	
Age—y	50 (40, 59)	55 (47, 61)	0.020
BMI *—Kg/m^2^	25.34 (23.03, 27.55)	25.95 (23.87, 28.39)	0.340
HBP *	13 (20.00)	205 (71.18)	<0.001
DM *	4 (6.15)	17 (5.90)	0.942
CHD *	3 (4.62)	27 (9.38)	0.214
Smoking	13 (20.00)	106 (36.81)	0.010
Alcohol	7 (10.77)	53 (18.40)	0.139

Data are presented as *n* (%), or median (interquartile range). BMI *, Body mass index; HBP *, High blood pressure; DM *, Diabetes mellitus; CHD *, Coronary heart disease.

**Table 2:** Characteristics associated with laboratory results.

Variables	TAD (*n* = 65)	NTAD (*n* = 288)	*p* Value
White blood cell count—10^9^/L	13.04 (10.43, 17.42)	11.28 (8.94, 13.90)	<0.001
Neutrophil percentage—%	83.41 (74.47, 87.53)	79.57 (73.26, 86.65)	0.156
Red blood cell count—10^12^/L	3.63 ± 0.32	4.34 ± 0.46	<0.001
Hemoglobin—g/L	112.53 (102.00, 130.00)	130.14 (117.50, 144.00)	<0.001
Fibrinogen—g/L	4.28 ± 1.36	4.88 ± 1.51	0.005
Total cholesterol—mmol/L	3.98 (3.31, 5.22)	4.63 (4.07, 5.31)	0.006
Triglyceride—mmol/L	1.32 (1.10, 1.60)	1.24 (0.93, 1.68)	0.153
HDL-C *—mmol/L	0.99 (0.88, 1.21)	1.14 (0.97, 1.48)	0.003
LDL-C *—mmol/L	2.34 ± 0.65	2.8 ± 0.52	0.001
Fasting glucose—mmol/L	7.10 (6.30, 7.60)	7.00 (6.20, 8.10)	0.930
Serum creatinine—μmol/L	64.22 (54.67, 77.26)	71.35 (60.38, 88.32)	0.013
Homocysteine—mmol/L	0.81 (0.73, 0.96)	0.99 (0.82, 1.23)	<0.001
Brain natriuretic peptide—pg/ml	73.29 (21.00, 145.50)	52.00 (28.00, 88.60)	0.698
C-reactive protein—mg/L	77.80 (40.60, 136.20)	82.60 (42.24, 126.16)	0.731
D-dimer—mg/L	10.51 (4.60, 18.20)	4.44 (2.29, 9.16)	<0.001
ESR *—mm/h	33.00 (22.00, 36.50)	28.00 (15.00, 45.00)	0.808

Data are presented as *n* (%), mean ± standard deviation, or median (interquartile range). HDL-C *, High-density lipoprotein cholesterol; LDL-C *, Low-density lipoprotein cholesterol; ESR *, Erythrocyte sedimentation rate.

**Table 3:** Characteristics of aortic dissection in radiology.

Variables	TAD (*n* = 65)	NTAD (*n* = 288)	*p* Value
Aortic arch branching type			
Type I	45 (69.23)	138 (47.92)	0.093
Type II	16 (24.62)	97 (33.68)	0.075
Type III	4 (6.15)	53 (18.40)	0.008
The length of dissection—mm	30.00 (22.00, 40.00)	344.00 (237.25, 400.00)	<0.001
The proximal zone of the involved aorta			0.228
Zone 3	61 (93.85)	277 (96.18)	
Zone 4	2 (3.08)	9 (3.13)	
Zone 5	2 (3.08)	2 (0.69)	
The distal zone of the involved aorta			<0.001
Zone 3	30(46.15)	9 (3.13)	
Zone 4	17(26.15)	11(3.82)	
Zone 5	8(12.31)	17(5.90)	
Zone 6	2 (3.08)	14(4.86)	
Zone 7	2 (3.08)	24 (8.33)	
Zone 8	4 (6.15)	43 (14.93)	
Zone 9	1 (1.54)	35(12.15)	
Zone 10	1 (1.54)	59(20.49)	
Zone 11	0 (0.00)	76(26.39)	
Maximum diameter of AA *—mm	36.86 ± 4.75	40.19 ± 4.81	<0.001
AA * > 40mm	13 (20.00)	141 (48.96)	<0.001
Maximum diameter of TL * + FL *—mm	35.80 (32.10, 39.80)	37.70 (34.00, 42.02)	0.013
Maximum diameter of TL *—mm	22.40 (16.6, 26.7)	19.10 (15.47, 23.92)	0.006
Maximum diameter of FL *—mm	16.40 (11.60, 21.50)	17.75 (14.40, 23.50)	0.037
Number of dissection process-involved zones	1.5 (1, 2)	8.0 (5, 9)	<0.001
PET * at the lesser curvature	29 (44.62)	69 (23.96)	0.001
Multiple entry tears	2 (3.08)	85 (29.51)	<0.001
Distance of PET from LSA *—mm	17.00 (10.00, 22.00)	20.00 (15.00,25.00)	0.244
Retrograde dissection	18 (27.69)	47 (16.32)	0.050
Dissecting aneurysms	5 (7.69)	38 (13.19)	0.271
Complicated aortic dissection	0	21(7.29)	0.001
Pleural effusions	0	8 (2.78)	
Bowel ischemia	0	6 (2.08)	
Kidney ischemia	0	6 (2.08)	
Lower extremity ischemia	0	5(1.74)	

Data are presented as *n* (%), mean ± standard deviation, or median (interquartile range). AA *, ascending aorta; TL *, true lumen; FL *, false lumen; PET *, primary entry tear; LSA *, left subclavian artery.

**Table 4:** Characteristics during procedure.

Variables	TAD (*n* = 65)	NTAD (*n* = 288)	*p* Value
Hospital stay—d	19 (14, 25)	19 (16, 25)	0.435
Duration before surgery—d	7.00 (2.00, 14.00)	11.00 (8.00, 15.00)	<0.001
Postoperative hospital stay—d	8.00 (6.00, 15.00)	8.00 (6.00, 11.00)	0.453
Emergency TEVAR	4 (6.15)	14 (4.86)	0.685
Operative time—h	1.97 ± 0.54	2.12 ± 0.43	0.143
Stent length—mm	160.00 (150.00, 180.00)	180.00 (160.00, 200.00)	<0.001
Stent diameter—mm	32.00 (28.00, 34.00)	32.00 (30.00, 34.00)	0.020
Chimney	2 (3.08)	16 (5.56)	0.545
Fenestration	0 (0.00)	5 (1.74)	0.589
LSA * coverage	12 (18.46)	66 (22.92)	0.538
Number of stents implanted			0.608
1	60 (92.31)	257 (89.24)	
2	5 (7.69)	31 (10.76)	

Data are presented as *n* (%), mean ± standard deviation, or median (interquartile range). LSA *, Left Subclavian Artery.

**Table 5:** Complications between the TAD and NTAD.

Variables	TAD (*n* = 65)	NTAD (*n* = 288)	*p* Value
During the perioperative period	14 (21.54)	66 (22.91)	0.849
Kidney ischemia	1 (1.54)	2 (0.69)	
SIRS * (death)	2 (3.08)	1 (0.35)	0.033
Endoleak	0	34 (11.80)	
Puncture site hematoma	4 (6.15)	29 (10.07)	
Deep vein thrombosis	5 (7.69)	0	
Pneumonia	2 (3.08)	0	
>30 days	0	38 (13.19)	0.004
SINE *	0	2 (0.69)	
Pseudoaneurysm	0	4 (1.39)	
Retrograde dissection	0	3 (1.04)	
Upper limb ischemic	0	2 (0.69)	
Lower extremity ischemia	0	3 (1.04)	
Kidney ischemia	0	2 (0.69)	
Bowel ischemia	0	3 (1.04)	
Distal segmental aortic enlargement **	0	19 (6.61)	

Data are presented as *n* (%). SIRS *, systemic inflammatory response syndrome; SINE *, distal stent-graft-induced new entry. ** The definition of DSAE (Distal segmental aortic enlargement) is as follows: postoperative dilation of the distal aorta, local aneurysmal dilation of the aorta, with a diameter 1.5 times that of a normal aorta, or an increase in aortic diameter > 10 mm/year.

**Table 6:** Outcome between TAD and NTAD.

Outcome	TAD	NTAD	HR (95% CI)	OR (95% CI)	*p* Value
Complication			-	0.49 [0.26, 0.92]	0.027
No	51 (78.46)	184 (63.89)			
Yes	14 (21.54)	104 (36.11)			
Death			0.38 [0.08,1.68]	-	0.203
No	63 (96.92)	266 (92.36)			
Yes	2 (3.08)	22 (7.64)			

Data are presented as *n* (%).

## Data Availability

The corresponding author can provide access to the data used in this study if needed.

## Supplementary Materials

The following supporting information can be downloaded at: https://www.mdpi.com/article/10.3390/jcm12247535/s1, Table S1: The causes and main manifestations of trauma in patients.
