# Supplementary material for: Traumatic Aortic Dissection as a Unique Clinical Entity: A Single-Center Retrospective Study
Source: J Clin Med. 2023 Dec 6;12(24):7535. doi: 10.3390/jcm12247535 (PMC10744057; doi:10.3390/jcm12247535)
Supplement: Supplementary file 1 [file jcm-12-07535-s001.zip › jcm-2697986-supplementary.pdf]

Supplementary Table S1. The causes and main manifestations of trauma in patients.

Number	Gender	Age	Trauma representation
1	Man	52	hip fracture, facial injuries
2	Man	52	lower extremity fracture
3	Man	53	lower extremity fracture, rib fracture
4	Woman	49	rib fracture, splenic rupture
5	Man	49	rib fracture
6	Woman	72	hip fracture, rib fracture
7	Man	68	rib fracture, fracture of lumbar vertebra, pneumothorax
8	Man	49	rib fracture, rupture of small intestine, splenic rupture, craniocerebral trauma
9	Man	27	rib fracture, hepatic blunt trauma, splenic rupture, fracture of upper limb, fracture of lumbar vertebra, ARDS
10	Man	53	rib fracture, splenic rupture
11	Woman	49	fracture of malleolus
12	Man	49	traumatic shock, rib fracture, fracture of pelvis, contusion of kidney, contusion of lung
13	Woman	48	lower extremity fracture, rib fracture
14	Man	65	lower limb fracture, pulmonary contusion, rib fracture, fracture of thoracic vertebrae
15	Man	83	pelvic fracture, rib fracture
16	Woman	70	rib fracture, lower extremity fracture
17	Man	57	Cervical sprain, rib fracture, clavicle fracture
18	Man	41	soft tissue contusion
19	Man	41	rib fracture, pulmonary contusion
20	Man	71	rib fracture, lower extremity fracture
21	Man	36	ARDS, pneumothorax, lower extremity fracture
22	Man	27	infarct of kidney, infarct of spleen, rib fracture
23	Woman	34	rib fracture, lower extremity fracture

24	Man	43	rib fracture, infarct of spleen
25	Man	60	rib fracture, lower extremity fracture
26	Man	40	rib fracture
27	Man	70	rib fracture, lumbar vertebra fracture
28	Man	39	infarct of spleen
29	Man	35	pelvic fracture, lower extremity fracture, rib fracture
30	Man	50	rib fracture, liver contusion,
31	Man	34	haemorrhagic shock, infarct of spleen, lower extremity fracture
32	Woman	59	haemorrhagic shock, upper limb fracture, lower extremity fracture, fracture of lumbar vertebra
33	Man	38	Cervical Spinal Fractures, fracture of clavicle, rib fracture, thoracic fractures
34	Man	64	rib fracture, pulmonary contusion, compression fracture of the thoracic, lower extremity fracture vertebra
35	Man	56	rib fracture, infarct of spleen
36	Man	55	rib fracture
37	Man	61	rib fracture, lower extremity fracture
38	Man	46	rib fracture, lumbar vertebrae fracture
39	Man	50	rib fracture
40	Man	51	rib fracture
41	Man	55	rib fracture, infarct of spleen
42	Woman	63	ARDS, lower extremity fracture, pelvic fracture
43	Woman	54	haemorrhagic shock, upper limb fracture, lower extremity fracture, fracture of lumbar vertebra
44	Man	37	infarct of spleen, rib fracture, Fracture of facial bone
45	Man	49	haemorrhagic shock, upper limb fracture, lower extremity fracture, fracture of lumbar vertebra
46	Man	52	rib fracture
47	Man	78	lower extremity fracture, pelvic fracture, rib fracture

48	Man	34	infarct of spleen
49	Man	61	rib fracture, lumbar vertebrae fracture
50	Man	65	haemorrhagic shock, upper limb fracture, lower extremity fracture, fracture of lumbar vertebra
51	Man	59	rib fracture, infarct of spleen
52	Man	31	pulmonary contusion, pneumothorax, rib fracture,
53	Woman	50	pelvic fracture, rib fracture, lower extremity fracture
54	Man	67	scapular fracture, clavicle fracture, rib fracture, pneumothorax
55	Man	35	lower extremity fracture, rib fracture, pulmonary contusion
56	Man	51	rib fracture, pulmonary contusion
57	Man	30	pulmonary contusion, rib fracture
58	Man	35	upper limb fracture, lower extremity fracture, lumbar vertebrae fracture
59	Man	30	rib fracture, lower extremity fracture
60	Man	53	upper limb fracture, lower extremity fracture, fracture of lumbar vertebra
61	Woman	43	pulmonary contusion, pelvic fracture
62	Man	50	pneumothorax, rib fracture
63	Man	48	upper limb fracture, lower extremity fracture
64	Man	34	rib fracture, pulmonary contusion
65	Woman	48	lower extremity fracture, rib fracture
